# A Machine Learning-Based Diagnostic Nomogram for Moyamoya Disease: The Validation of Hypoxia-Immune Gene Signatures

**DOI:** 10.3390/bioengineering12060577

**Published:** 2025-05-27

**Authors:** Cunxin Tan, Xilong Wang, Zhenyu Zhou, Yutong Liu, Shihao He, Yuanli Zhao

**Affiliations:** 1 Department of Neurosurgery, Peking University International Hospital, Beijing 102206, China; tancunxin@pkuih.edu.cn; 2 Department of Neurosurgery, Peking Union Medical College Hospital, Peking Union Medical College and Chinese Academy of Medical Sciences, Beijing 100730, China; yutong-l22@mails.tsinghua.edu.cn; 3 Department of Neurosurgery, Beijing Tiantan Hospital, Capital Medical University, Beijing 100070, China; 1801060@ccmu.edu.cn (X.W.); zhouzy300@163.com (Z.Z.)

**Keywords:** moyamoya disease, machine learning, nomogram, hypoxia-immune gene

## Abstract

Moyamoya disease (MMD) is a cerebrovascular disease which can result in severe strokes. However, its etiology is still unknown. We analyzed gene expression datasets from 36 MMD patients and 24 controls to identify differentially expressed genes. Using weighted gene co-expression network analysis and databases such as KEGG, we identified hypoxia-immune-related genes. These genes were further refined through machine learning algorithms. The diagnostic value was confirmed using an external dataset, and a diagnostic nomogram was constructed. Additionally, gene set enrichment analysis was conducted, and a competitive endogenous RNA (ceRNA) network was built to predict potential therapeutic targets. Our study identified *AKT1*, *CLDN3*, *ISG20*, and *TGFB2* as the key hypoxia-immune genes associated with MMD. These genes were implicated in epithelial–mesenchymal transition, angiogenesis, and cell adhesion, suggesting a role in MMD pathogenesis. Further, our study constructed the ceRNA network and predicted potential drug targets for MMD. We obtained the top 10 drugs in the interaction of the four key genes that might serve as potential targets for the treatment of MMD. In conclusion, this study comprehensively analyzes the role of hypoxia-immune genes in MMD, which is conducive to the development of new diagnostic and therapeutic approaches and the exploration of the potential pathogenesis of MMD.

## 1. Introduction

Moyamoya disease (MMD) is a rare type of cerebrovascular disorder characterized by the chronic narrowing or occlusion of the intracranial terminal internal carotid arteries and abnormal angiogenesis at the base of the skull, which causes cerebral hemodynamic disturbances that cause cerebral ischemia and hypoxia of brain tissue, eventually leading to a variety of severe clinical symptoms, including aphasia, epilepsy, cognitive impairment, muscle weakness, and even stroke [1]. Unfortunately, the etiology underlying MMD remains unclear. As a result, there exists a dearth of definitive diagnostic methodologies and therapeutic approaches tailored specifically for MMD [1,2].

A number of research endeavors have suggested the potential involvement of genetic factors in the pathogenesis of MMD [2,3,4]. And *RNF213*, located in the 17q25-ter region, has been reported to be closely associated with MMD, which has provided a new perspective to further explore diagnosis and treatment strategies [2]. Besides genetic factors, microenvironmental and immune-related factors may also be associated with MMD [5]. Recently, there was a study finding that immune dysregulation existed in patients with MMD, which suggested a potential association between the pathogenesis of MMD and immune system [6]. In summary, genetic and immune-related factors likely play a role in the mechanisms of MMD’s pathophysiology.

In an immunohistochemical study, the overexpressed hypoxia-inducing factor-1alpha (HIF-1a) has been found in the endothelium and intima of the middle cerebral arteries of MMD patients [7]. Moreover, the signaling pathway mediated by HIF-1a has been identified as a pivotal factor contributing to the angiogenesis in MMD [8]. In addition, there was an in vivo experiment in mice that suggested that *RNF213* R4810K, which is the frequently observed variant in east Asian MMD patients, can inhibit angiogenesis induced by the conditions of hypoxia [9]. These results also serve as a reminder of the potential relevance of hypoxia and hypoxia phenotype-related genes (HPRs) to MMD.

Currently, the genetic background associated with the immunity and hypoxia of MMD has been poorly studied. In order to find appropriate diagnostic and therapeutic targets, we identified the feature hypoxia-immune genes of MMD by combining bioinformatics methods including immune infiltration, high-throughput sequencing, and machine learning, and we validated it with an additional external dataset in this study.

## 2. Materials and Methods

### 2.1. Data Acquisition

The National Center for Biotechnology Information Gene Expression Omnibus (NCBI GEO, https://www.ncbi.nlm.nih.gov/ (accessed on 12 August 2023)) is a microarray and high-throughput sequencing database. We downloaded the datasets from the NCBI GEO database for the training set according to the following inclusion criteria: (1) the data of MMD are independent expression profiles; (2) the datasets include both MMD and control profiles; (3) the specimens in these datasets are derived from human tissue. According to the inclusion criteria, the data files from the series matrix of GSE189993, GSE157628, and GSE141024 including 36 MMD patients and 24 controls, in which there were 21, 11, and 4 MMD patients, respectively, and 11, 9, and 4 controls, respectively, were downloaded. And the GPL16699 file was used to annotate all of the series matrix data files.

The validation set consisted of self-test data derived from superficial temporal artery (STA) biopsy specimens from 10 patients with MMD and 3 controls. The collection of bio-specimens and data was approved by the Institutional Ethics Committee of Peking Union Medical College Hospital, Beijing, China (I-24PJ2435), and all specimens were obtained with written informed consent.

During surgery, all STA tissue samples were carefully collected using sterile instruments. Immediately after extraction, the samples were placed in pre-chilled, RNase-free microcentrifuge tubes and promptly immersed in liquid nitrogen to prevent RNA degradation. The frozen samples were then transferred to a −80 °C freezer for long-term storage until further processing and transported using dry ice as the cooling medium. RNA extraction from surgical samples of the STA was performed using the RNAprep Pure Micro Kit (DP420, Tiangen Biotech, Beijing, China). RNA integrity was assessed using the RNA Nano 6000 Assay Kit of the Bioanalyzer 2100 system (Agilent technologies, Santa Clara, CA, USA). The total RNA extracted from the aforementioned STA samples was used as input material for the RNA sample preparations. The amplification of the extracted RNA followed the SMART-Seq V4 Ultra Low Input RNA Kit protocol (634888, Takara, Kusatsu, Japan). The library was subsequently prepared following the NEBNext Ultra Directional RNA Library Prep Kit (Illumina) instructions. Sequencing was conducted on the Illumina NovaSeq 6000 platform. After sequencing, quality control checks were conducted, including assessments of error rate and GC content distribution. Raw data in fastq format were initially processed using in-house Perl scripts. During this step, clean data (clean reads) were obtained by eliminating reads containing adapters, reads with poly-N, and low-quality reads (where the number of bases with Qphred ≤ 20 accounts for more than 50% of the read length) from the raw data. Concurrently, the Q20, Q30, and GC content of the clean data were calculated. All downstream analyses were based on these clean data of high quality.

The datasets of HPRs were downloaded from reputable databases, including the Kyoto Encyclopedia of Genes and Genomes (KEGG, https://www.kegg.jp/pathway/hsa04066 (accessed on 12 August 2023)), the Molecular Signatures database (MSigDB, http://www.gseamsigdb.org/gsea/msigdb/cards/HALLMARK_HYPOXIA (accessed on 12 August 2023)), and the Universal Protein Resource (UniProt, https://www.uniprot.org/help/uniprotkb (accessed on 12 August 2023)). Specifically, we utilized the KEGG database to compile an inventory of HPRs associated with hypoxia-inducing factor-1 signaling pathway (*n* = 109). In addition, we incorporated the Hallmark hypoxia gene set using the MSigDB database (*n* = 200) and downloaded hypoxia-associated genes from the UniProt database, which were included in the Human Disease Spectrum.

### 2.2. Data Processing

The raw data of the training set were firstly processed using the “ArrayExpress” package (version 1.62.0, https://www.bioconductor.org/packages/release/bioc/html/ArrayExpress.html (accessed on 12 August 2023)) in R software (version 4.2.2) to perform background correction and standardization of the expression matrix and annotate the genes. Then, the expression matrix file of the mRNA probe corresponding to each dataset and the corresponding annotation file were downloaded, and the probes were transformed into gene symbols one by one, in which those unmatched probes were removed. For different probes mapped to the same gene, the average values were utilized as the representative expression levels for those genes. The Surrogate Variable Analysis (SVA) method is designed to identify and correct for hidden confounders in transcriptomic data [10,11]. Subsequently, the “SVA” package (version 3.50.0, http://www.bioconductor.org/packages/release/bioc/html/sva.html (accessed on 12 August 2023)) in R software was performed to remove the batch effects of GSE189993, GSE157628, and GSE141024 expression matrices and to merge these matrices to obtain the training set [11].

For the validation set, the self-test raw expression data were normalized using log(x+1) transformations, while genes that expressed 0 in more than 50% of the samples were eliminated.

### 2.3. Gene Differential Expression Analysis

The “limma” package (version 3.58.1; https://bioconductor.org/packages/release/bioc/html/limma.html (accessed on 12 August 2023)) in R software was utilized to calculate *p*-value and log2FoldChange (log2FC) to analyze differences between MMD and control groups [12]. The criteria for identifying differentially expressed genes (DEGs) were |log2FC| ≥ 0.2 and *p* < 0.05 [13].

### 2.4. Analysis of Immune Cell Infiltration

Single-sample gene set enrichment analysis (ssGSEA) is widely utilized for the evaluation of immune cell types in a microenvironment. We utilized the ssGSEA algorithm in the “GSVA” package (version 1.50.5, https://www.bioconductor.org/packages/release/bioc/html/GSVA.html (accessed on 12 August 2023)) in R software to calculate the proportion of twenty-eight kinds of immune cells in the training set [14]. Then, we employed the Wilcoxon signed-rank test (Wilcoxon test) to examine the difference in immune cell distribution between MMD and control groups, and the Pearson correlation coefficient was then used for the association between each immune cell.

### 2.5. Immunohistochemistry Staining

The frozen STA specimens from MMD patients were taken out, allowed to stand at room temperature for 10 min, and then transferred to a 4 °C refrigerator to thaw overnight. Temporal STA samples from MMD patients were fixed in 10% neutral-buffered formalin at room temperature for 24 h. The tissues were then dehydrated, embedded in paraffin, and sectioned into 4 µm thick slices using a microtome. For immunohistochemistry, the sections were deparaffinized in xylene and rehydrated through a graded ethanol series. Antigen retrieval was performed by heating the sections in citrate buffer (pH 6.0) at 95 °C for 20 min. To block endogenous peroxidase activity, the sections were incubated with 3% hydrogen peroxide for 10 min at room temperature. Non-specific binding was minimized by blocking with 5% bovine serum albumin (BSA) for 30 min. The sections were incubated overnight at 4 °C in a humidified chamber with a primary antibody against CD56 (1:500, #ab75813, Abcam, Cambridge, UK). After washing, the slides were incubated with a horseradish peroxidase (HRP)-conjugated secondary antibody for 1 h at room temperature. Signal development was carried out using a diaminobenzidine (DAB) substrate kit, and the sections were counterstained with hematoxylin. The slides were then dehydrated, cleared, and mounted for imaging.

### 2.6. Weighed Gene Co-Expression Network Analysis

Weighed gene co-expression network analysis (WGCNA) was used to find the gene clusters that exhibit a high degree of correlation with the sample phenotype in high-throughput data. Initially, it was presumed that the gene network adhered to the principles of a scale-free network. Subsequently, the gene co-expression correlation matrix and the adjacency function derived from the gene network were precisely defined. Following this, the varying coefficients of distinct nodes were computed to pinpoint the modules composed of gene clusters that are linked to the phenotype [15]. We used the “WGCNA” package (version 1.72-5) in R software to screen immune-related genes by analyzing the matrix data of DEGs with differential immune cell expression levels of the training set as traits [15].

### 2.7. Identification of Hypoxia-Immune Genes

The data of HPRs downloaded from KEGG, MSigDB, and UniProt were de-emphasized to obtain the HPRs (*n* = 710) [16,17]. Then, we took the intersection of the HPRs with the immune-related genes obtained by WGCNA to acquire the hypoxia-immune genes for subsequent analysis.

### 2.8. Construction of Protein–Protein Interaction Network

The STRING database (https://cn.string-db.org/ (accessed on 12 August 2023)) contains known and predicted protein–protein interactions (PPIs). In this study, STRING was employed to retrieve interactions between gene product proteins of hypoxia-immune genes, and a PPI network was constructed with a threshold of combined score > 0.4.

### 2.9. Machine Learning Screening for Diagnostic Feature Genes

The least absolute shrinkage and selection operator (LASSO) is a dimensionality reduction method that has shown advantages when assessing high-dimensional datasets. LASSO analysis uses regularized penalty parameters to select feature variables, utilizing 10-fold cross-validation [18]. The random forest (RF) method outperforms linear discriminant analysis and mean square error methods in the selection of pertinent features and removing redundant ones. The selection of key genes in RF algorithms was executed using 10-fold cross-validation, and those genes exhibiting a Mean-Decrease-Gini value > 3 were selected as significant variables [19]. Support vector machine–recursive feature elimination (SVM-RFE) is a supervised machine learning method which can rank the feature genes associated with MMD and assess predictive performance by 10-fold cross-validation to identify characterized genes [20].

In this study, the three machine learning algorithms, LASSO, RF, and SVM-RFE, were implemented using the corresponding R packages: “glmnet”, “randomForest”, and “e1071” [18,19,20]. The intersection of the outcomes from the three algorithms was regarded as comprising the key diagnostic genes.

### 2.10. Validation and Performance Evaluation of Characteristic Genes

Employing Wilcoxon tests, the distinctions in key diagnostic genes among diverse states of MMD were contrasted, drawing upon the datasets from the training and validation sets. And the receiver operating characteristic (ROC) curve and area under curve (AUC) were plotted for both the training and validation sets utilizing the “pROC” package (version 1.18.5, http://expasy.org/tools/pROC/ (accessed on 12 August 2023)) in R software to assess the efficacy of the diagnostic feature genes [21].

### 2.11. Diagnostic Nomogram Visualization

The nomogram fundamentally represents a graphical depiction of the regression equation. It creates a scale that corresponds to the magnitude of the regression coefficients for all independent variables. Each value level of each independent variable is assigned a score, allowing for the calculation of a total score for each sample. Subsequently, the probability of each sample’s occurrence is determined through a conversion function that correlates the total score with the probability of the endpoint events. The calibration curves, ROC curves, and decision curve analysis (DCA) of clinical prediction models can assess the predictive capacity of the nomogram. In this study, we constructed the diagnostic nomogram for MMD using the “rms” package (version 6.8-0, https://cran.r-project.org/web/packages/rms/index.html (accessed on 12 August 2023)) in R software [22].

### 2.12. Association Analysis of Immune Cells and Feature Genes

The ssGSEA algorithm in the “GSVA” package in R software was used to calculate the scores of twenty-eight kinds of immune cells. The Pearson correlation coefficient was then used for the association between immune cells and key diagnostic genes.

### 2.13. Gene Set Enrichment Pathway Analysis

Gene set enrichment pathway analysis (GSEA) is a computational method used to assess whether a set of genes shows a statistically significant difference between two biological states [23]. In this study, GSEA was used to analyze the KEGG enrichment involved in key diagnostic genes. The adj. p corrected by the Benjamini–Hochberg method < 0.05 and absolute normalized enrichment score (NES) value > 1.0 were set as cutoff threshold criteria.

### 2.14. Prediction of Potential Drugs

The Drug Signatures Database (DSigDB, https://dsigdb.tanlab.org/DSigDBv1.0/ (accessed on 12 August 2023)) is a new resource of gene sets linking drugs or compounds and their target genes. It currently comprises 22,527 gene sets, which include 17,389 unique compounds and span across 19,531 genes. In this study, we examined the correlation between key diagnostic genes and drugs through the DSigDB.

### 2.15. Construction of Competing Endogenous RNA (ceRNA) Network

Regarding regulatory networks that aim to target specific hypoxia-immune genes, we acquired differentially expressed microRNAs (miRNAs) and long non-coding RNAs (lncRNAs) from the self-test data, utilizing a threshold of |log2FC| > 1 as the criterion. Subsequently, we employed the miRWalk website (http://mirwalk.umm.uni-heidelberg.de/ (accessed on 12 August 2023)) to discern those miRNAs associated with the key hypoxia- and immune-related genes. We intersected the identified miRNAs with the set of differentially expressed miRNAs, thereby filtering out those miRNAs that showed opposite expression tendencies to the key hypoxia-immune genes. Similarly, utilizing correlation analysis, we predicted the lncRNAs that are associated with miRNAs in identical relational pairs. Subsequently, we identified and retained those lncRNAs which demonstrate an expression trend that is diametrically opposed to the key miRNAs previously mentioned. Finally, the regulatory network was visualized using Cytoscape (version 3.8.2, https://cytoscape.org/ (accessed on 12 August 2023)) [24].

### 2.16. Statistical Analysis

All statistical analyses were conducted utilizing R software (version 4.2.2), with the threshold of statistical significance set at *p* < 0.05.

## 3. Results

### 3.1. Identification of DEGs and WGCNA Immune-Related Genes

**Figure 1:**
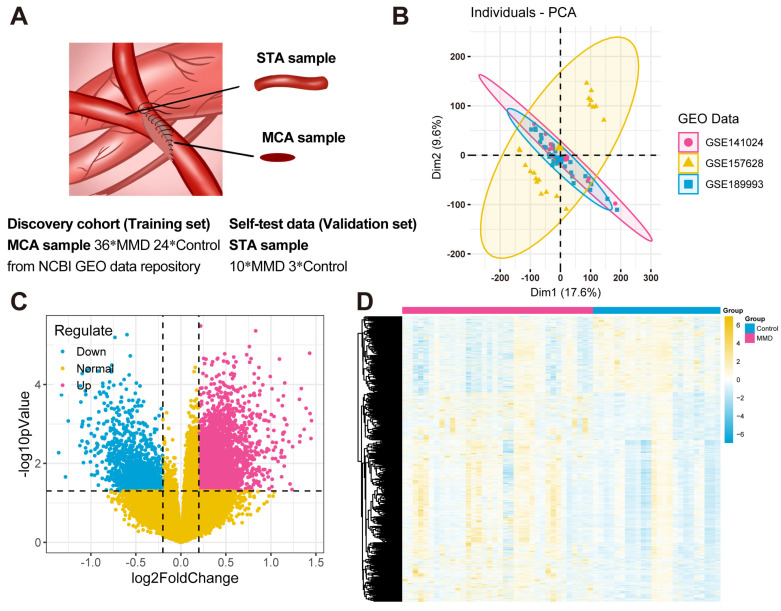
**Data acquisition and differentially expressed gene identification.** (**A**) In this study, the samples of the training set downloaded from the NCBI GEO database were mainly obtained from the middle cerebral arteries (MCAs) of 36 MMD patients and 24 controls. The samples of the validation set were obtained from the superficial temporal arteries (STAs) of 10 MMD patients undergoing bypass surgeries and 3 controls who underwent cerebral excision procedures because of epilepsy. All of them signed the informed consent forms. (**B**) The PCA plots of the results of batch effects processing. (**C**) A volcano plot illustrates the DEGs. Specifically, blue dots depict the down-regulated genes within the MMD group, while red dots illustrate the up-regulated genes in the same group. Additionally, yellow dots are utilized to signify the non-DEGs. (**D**) A heat map visually illustrates the expression patterns of DEGs. Within this heat map, each distinct column represents an individual sample, while each horizontal row signifies a DEG.

After background correction and standardization, there were a total of 36 MMD samples and 24 control samples included in the GSE189993, GSE157628, and GSE141024 datasets composing the training set. And the validation set consisted of 10 MMD patients and 3 controls. The sample information contained in this study is shown in Figure 1A. The SVA package was utilized to eliminate batch effects present in the series matrix data. Principal Component Analysis (PCA) was used for the presentation of the before and after batch processing results (Figure 1B) [25,26]. The processing significantly reduced the batch effects between datasets.

Next, the “limma” package was utilized to ascertain the DEGs in the comparison between the MMD and control groups (Figure 1C,D). A total of 4660 DEGs were identified, comprising 3416 up-regulated and 1244 down-regulated genes.

**Figure 2:**
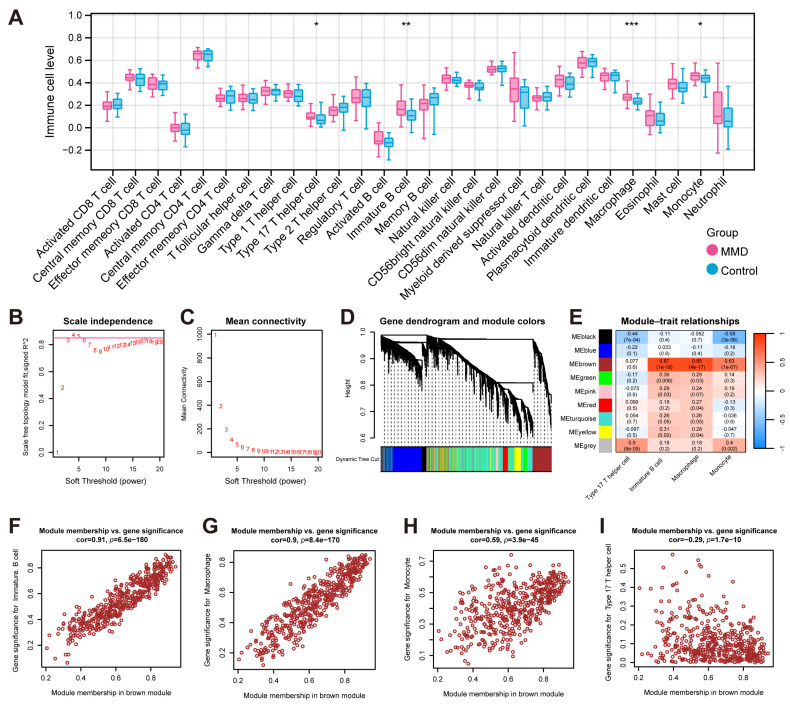
**The identification of immune-related genes.** (**A**) The results of ssGSEA, which show the comparison of immune cells’ infiltration between the MMD and control groups. (**B**) The plot for selecting the adjacency matrix weight parameter (power). The x-axis corresponds to the power values, while the y-axis depicts the squared correlation coefficient between log(k) and log(p(k)). Greater values of the squared correlation coefficient indicate that the network approximates the network-free scale distribution. The red line indicates the standard line where the squared correlation coefficient reaches a value of 0.85. (**C**) A schematic representation of the average connectivity of genes under different powers. The red points indicate the value of the average connectivity of the network nodes under the value of the power taken in part B. (**D**) A heatmap of the correlation of each module with the phenotype. Each column represents a module, and each row represents the correlation value. (**E**) The correlation between the traits (the horizontal axis) and the 7 modules (the vertical axis). (**F**) A scatterplot of the module memberships vs. significant phenotypic traits of immature B cells. (**G**) A scatterplot of the module memberships vs. significant phenotypic traits of macrophages. (**H**) A scatterplot of the module memberships vs. significant phenotypic traits of monocytes. (**I**) A scatterplot of the module memberships vs. significant phenotypic traits of type 17 T helper cells. * indicates that *p* is less than 0.05; ** indicates that *p* is less than 0.01, and *** indicates that *p* is less than 0.001.

Based on the expression profile data of the training set, the ssGSEA algorithm was employed to ascertain the disparities in the distribution levels of 28 distinct immune cell types between the MMD and control groups. (Figure 2A). There were significant differences in macrophage, immature B cell, monocyte, and type 17 T helper cell.

Finally, based on the expression DEGs, WGCNA was performed with differential immune cell expression levels obtained above as the phenotypic traits. In order to adhere to the prerequisites of scale-free network distribution, it is imperative to examine the parameter value of the adjacency matrix weight, denoted as “power”, define a spectrum of options for the parameters involved in network construction, and subsequently compute the topology matrix that reflects the scale-free distribution. We selected the power value at the point where the squared correlation coefficient first attained 0.85, which corresponds to a power of 4 (Figure 2B,C). Utilizing a combination of clustering and the dynamic pruning method, the genes exhibiting high correlation were clustered into distinct modules. Consequently, out of the 4660 genes analyzed, seven distinct modules were identified, with the exception of the gray module (Figure 2D). The subsequent analysis involved the computation of correlation coefficients between each module and the phenotypes (Figure 2E). It was determined that the brown module demonstrated the most pronounced correlation with immune cell-related phenotypes. And there was no significant correlation between the type 17 T helper cell traits and the brown module. Therefore, we chose the brown module as the focus and subsequently performed further association analysis of the brown module with the significant traits of immature B cells (Figure 2F), macrophages (Figure 2G), monocytes (Figure 2H), and type 17 T helper cells (Figure 2I). In total, 471 genes from the brown module were identified and utilized as immune-related module genes for subsequent analytical procedures.

### 3.2. The Hypoxia-Immune Genes Identification

**Figure 3:**
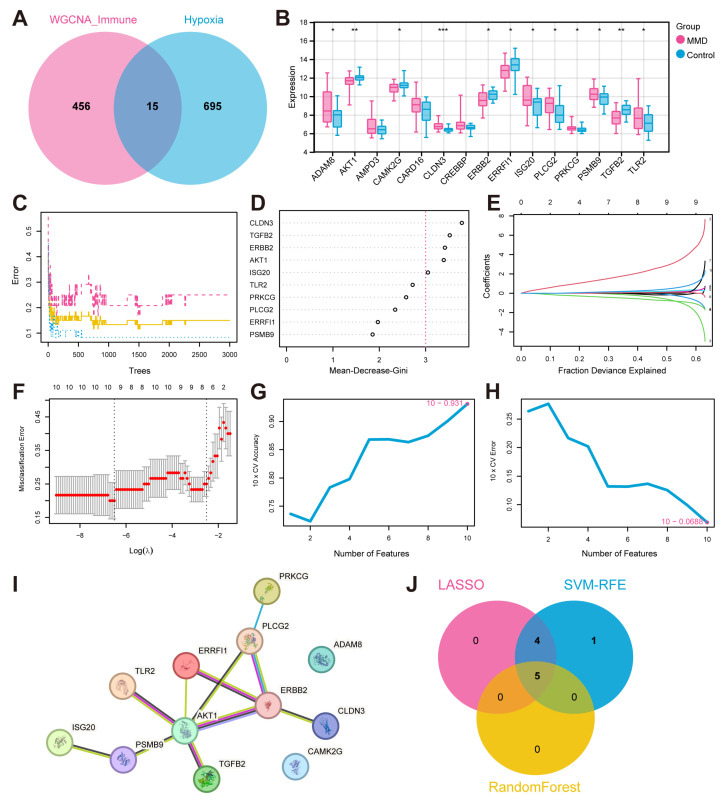
**The identification of feature hypoxia-immune genes and the construction of a PPI network.** (**A**) The Venn plot illustrates the intersection between WGCNA genes and hypoxia-related genes. (**B**) Differential expression box plots for 15 hypoxia-immune genes in the training set. FPKM: fragments per kilobase of exon model per million mapped fragments. (**C**) The RF model is utilized to explore the correlation between the number of trees and the error rates. (**D**) The genes arranged in accordance with their significance in the RF model. (**E**) Ten-fold cross-validation for optimizing parameter selection in the LASSO model. Each curve corresponds to a gene. (**F**) LASSO coefficient profiles. Solid vertical lines indicate partial likelihood deviations, and dashed lines are drawn vertically at the optimal lambda. (**G**,**H**) The results of the SVM-REF algorithm. (**I**) The PPI network plot. (**J**) A Venn plot of the intersection of characteristic genes. * indicates that *p* is less than 0.05; ** indicates that *p* is less than 0.01, and *** indicates that *p* is less than 0.001.

Based on the 471 immune-related genes identified by WGCNA and the 710 hypoxia related genes obtained from the KEGG, MSigDB, and UniProt databases, there were fifteen intersecting genes identified as hypoxia-immune genes (Figure 3A). Upon subsequent examination of the expression levels of the fifteen genes in the training set, it was determined that twelve of these genes displayed marked variations in expression between the MMD and control group. The genes in question were *ADAMB*, *AKT1*, *CAMK2G*, *CLDN3*, *ERBB2*, *ERRFI1*, *ISG20*, *PLCG2*, *PRKCG*, *PSMB9*, *TGFB2*, and *TLR2* (Figure 3B).

### 3.3. Key Gene Screening and PPI Network Establishing

We employed the STRING database for the aforementioned twelve hypoxia-immune genes that showed significant differences in the training set, with the minimum reciprocal activity set to 0.4. Among them, *ADAM8* and *CAMK2G* were not included in the network. The other ten key genes were further analyzed next (Figure 3I).

Next, we applied the machine learning algorithms to select characteristic genes among the ten key hypoxia-immune genes. The RF algorithm identified five feature genes of relative importance by Mean-Decrease-Gini > three (Figure 3C,D). Following a thorough 10-fold cross-validation procedure, the LASSO algorithm was employed to construct a classifier utilizing the minimum criterion that demonstrated high accuracy. Following a thorough 10-fold cross-validation procedure, the LASSO algorithm was employed to construct a classifier utilizing the criterion that demonstrated the highest accuracy. This resulted in the precise identification of a total of nine characteristic genes (Figure 3E,F). In the context of SVM-RFE, the classifier’s error rate was minimized when the number of features was ten (Figure 3G,H). After intersection, five characteristic genes common to the machine learning algorithms were finally identified, including *AKT1*, *CLDN3*, *ERBB2*, *ISG20*, and *TGFB2* (Figure 3J).

### 3.4. Expression Validation and Evaluation of Characteristic Genes

**Figure 4:**
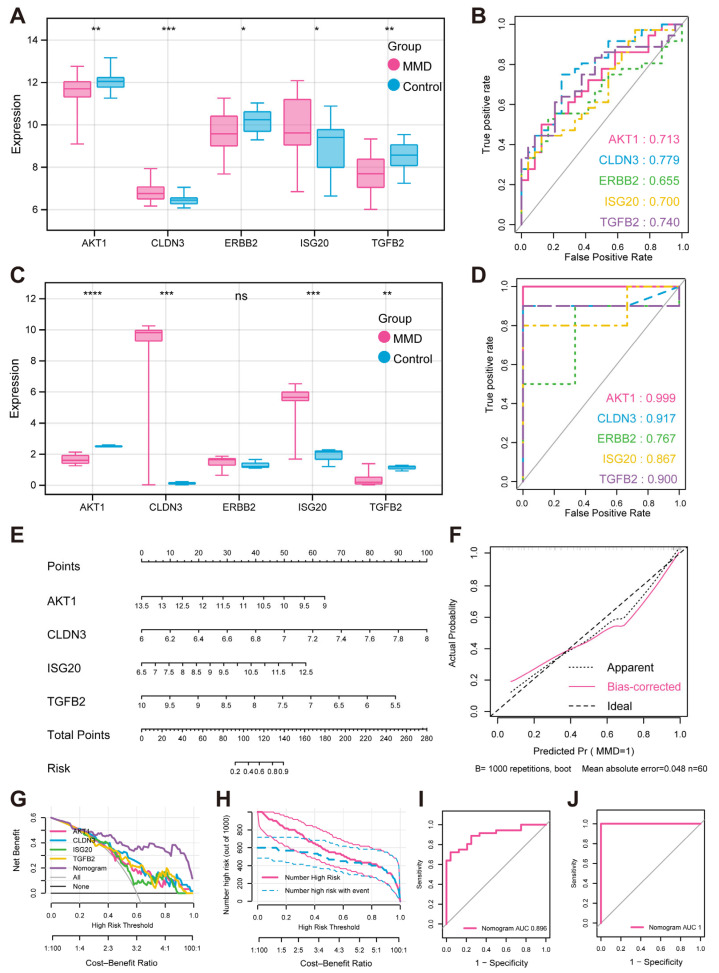
**Expression verification and performance evaluation of feature hypoxia-immune genes** (**A**) Box plots illustrate expression of feature genes in training set. (**B**) Predicted ROC curves of feature genes in training set (**C**). Expression box plots of feature genes in validation set. (**D**) Predicted ROC curves of feature genes in validation set. (**E**) MMD diagnosis nomogram. (**F**) Calibration curve for evaluating predictive ability of nomogram. (**G**) DCA curve for evaluating clinical value of nomogram. (**H**) Clinical impact curve of nomogram based on DCA curve. (**I**) ROC curve for evaluating predictive ability of nomogram in training set. (**J**) ROC curve for evaluating predictive ability of nomogram in validation set. * indicates that *p* is less than 0.05; ** indicates that *p* is less than 0.01, *** indicates that *p* is less than 0.001, **** indicates that *p* is less than 0.0001.

To validate the above five characteristic genes, we further analyzed their expression patterns within both the validation set and the training set (Figure 4A–D). The results indicated that there were significant disparities in the expression levels of the characteristic genes between the MMD and control groups, with the exception of *ERBB2*. Additionally, the ROC curves also revealed the exceptional predictive power of these genes.

To further assess the combined diagnostic capability of *AKT1*, *CLDN3*, *ISG20*, and *TGFB2* for MMD, we included them in the nomogram and used calibration curves to assess the predictive capability of the nomogram (Figure 4E,F). The results clearly demonstrated a negligible margin of error between the empirically observed risk of MMD disease occurrence and the risk estimated by the predictive nomogram. These findings underscored the exceptional predictive capability of the nomogram in accurately forecasting MMD. In addition, decision curve analysis (DCA) indicated that the “nomogram” curve outperformed the gray line curve, suggesting that the clinical efficacy of the MMD’s nomogram was more favorable (Figure 4G). In order to evaluate the clinical efficacy of the nomogram more intuitively, the clinical impact curve was constructed utilizing the DCA curve (Figure 4H). The “Number high risk” curve closely parallels the “Number high risk with event” curve when the range of high-risk threshold is from 0.8 to 1, indicating that the nomogram possesses ideal predictive ability. In the training set, the ROC curve shows that the nomogram was good at distinguishing between the presence or absence of MMD (Figure 4I). And in the validation set, the ROC curve also shows the nomogram’s specific and significant predictive performance (Figure 4J). Both AUC values were greater than 0.8, which indicates that the prediction performance of the nomogram was good.

### 3.5. Immune Cell Correlation Analysis and GSEA

**Figure 5:**
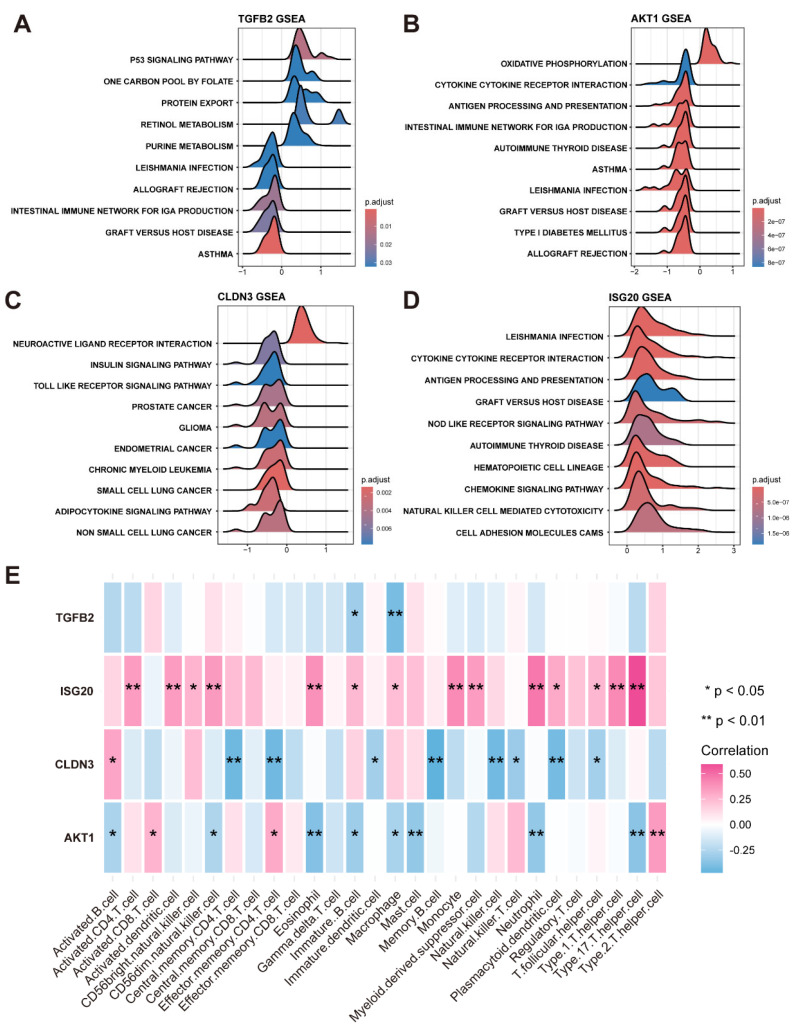
**Gene set enrichment pathway analysis and analysis of immune cell infiltration.** (**A**) Top 10 pathways of KEGG enrichment involving TGFB2. (**B**) Top 10 pathways of KEGG enrichment involving AKT1. (**C**) Top 10 pathways of KEGG enrichment involving CLDN3. (**D**) Top 10 pathways of KEGG enrichment involving ISG20. (**E**) Heatmap illustrates correlation between 4 feature genes and immune cells, where one asterisk denotes *p*-value less than 0.05, two asterisks indicate *p*-value value less than 0.01.

Employing the expression profile data derived from the training set, the correlation between the four feature genes and immune cells was calculated (Figure 5E).

In addition, based on KEGG datasets, the KEGG signaling pathways involved in the four feature genes were analyzed by GSEA, adhering to stringent thresholds of an adjusted *p*-value threshold of less than 0.05 and an NES with an absolute value exceeding 1 (Figure 5A–D).

### 3.6. Drug Prediction and ceRNA Network Construction

**Figure 6:**
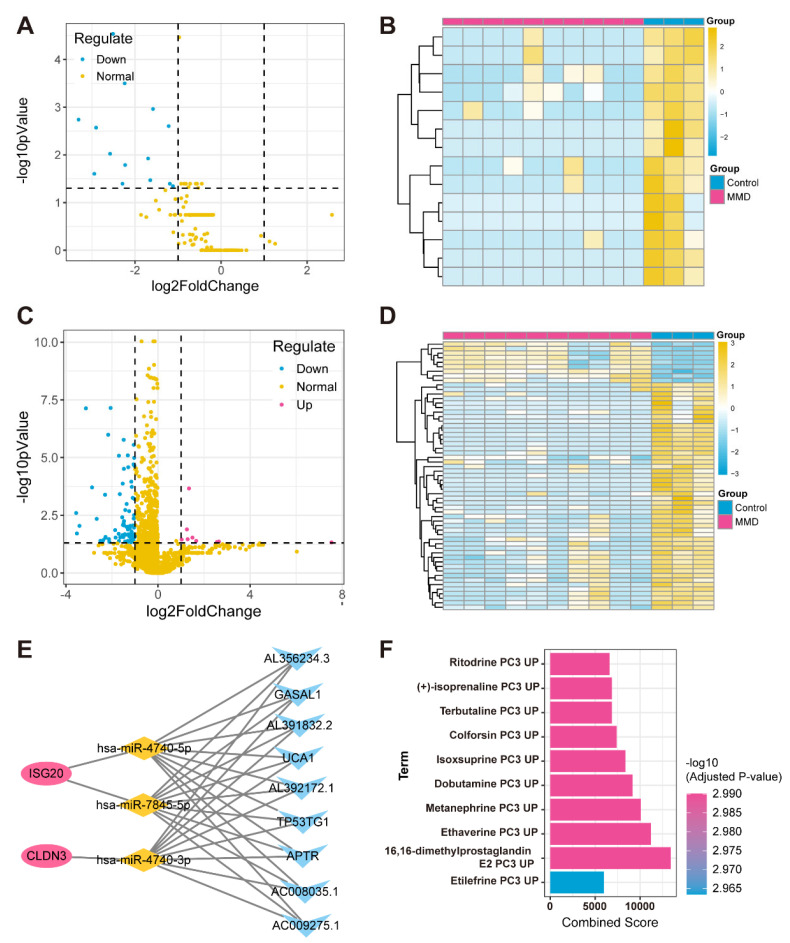
**Construction of ceRNA network for feature hypoxia-immune genes.** (**A**) Volcano plot depicts differential expression levels, with blue dots specifically highlighting down-regulated miRNAs in MMD group, red dots representing up-regulated miRNAs in MMD group, and yellow dots indicating normally expressed miRNAs. (**B**) Heatmap of differentially expressed miRNAs. (**C**) Volcano plot illustrates levels of differential expression. Blue dots represent down-regulated lncRNAs in MMD group. (**D**) Heatmap of differentially expressed lncRNAs. (**E**) ceRNA regulatory network in which red circles represent feature genes, yellow diamonds represent miRNAs, and blue triangles represent lncRNAs. (**F**) Bar graph of relationship between feature genes and drugs.

In the regulatory network targeting feature hypoxia-immune genes, we initially identified miRNAs and lncRNAs with significant expression differences between the MMD and control groups through differential expression analysis using the self-test data (Figure 6A–D). Subsequently, the miRWalk website was utilized to predict miRNAs associated with the feature hypoxia-immune genes. Next, we overlapped these predicted miRNAs with the differentially expressed miRNAs found by self-testing and screened for miRNAs (named key miRNAs) that showed opposite expression trends to the feature hypoxia-immune genes. Subsequently, the expression correlation between key miRNAs and differentially expressed lncRNAs was calculated using the Spearman correlation function (*p*-value < 0.05, and R < −0.6), thus obtaining the lncRNAs that have opposite and correlated expression trends to the key miRNAs (that is, those that are negatively correlated to the key miRNAs, named key lncRNAs) (Figure 6E).

In addition, we obtained drug information on four feature genes’ interactions through the DSigDB database and sorted them by adj. p from smallest to largest (Figure 6F).

### 3.7. Immunofluorescence Staining of STA from MMD Patients

**Figure 7:**
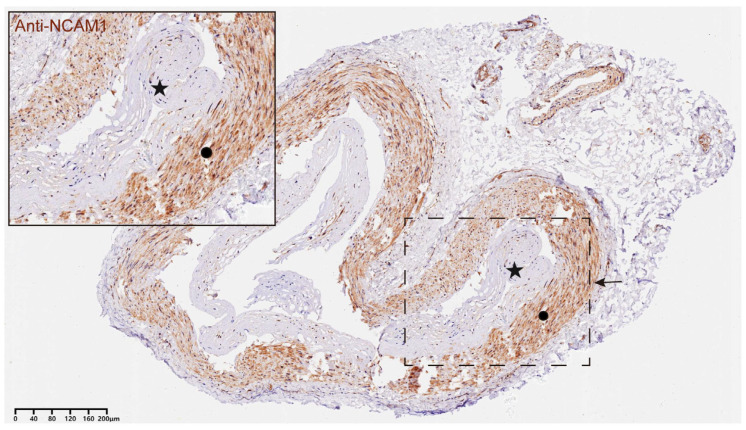
**Representative example of immunohistochemical images of CD56+ NKT cells in STA tissues from MMD patients.** A representative immunohistochemical image of CD56 in an STA sample from a patient with MMD. The star in the figure indicates the hyperplastic intima of the STA in MMD, and the circle refers to the media layer of STA. Positive CD56 staining is seen evident in both the intima and the media layer, suggesting that immune infiltration may be prevalent in STA from MMD patients. The region of interest is highlighted in the inset, and the magnified image emphasizes the localization of NKT cells within the vessel wall of the MMD sample. Scale bar = 40 μm.

CD56 (NCAM1), a member of the immunoglobulin superfamily, serves as a core marker for NK cells and is also frequently expressed in certain T cell subpopulations. We observed significant CD56-positive staining in both the endothelium and mesothelium of STA samples from MMD patients, but not in the controls, indicating the presence of immune cell infiltration in the STAs of MMD patients. Figure 7 shows the results of immunofluorescence staining in a patient with MMD.

## 4. Discussion

For MMD, the development of appropriate diagnostic and treatment strategies depends on the understanding of its etiology. Unfortunately, the pathogenesis of MMD remains largely unknown, which has led to the absence of specific experimental models and therapeutic drugs [27]. Currently, MMD is a diagnosis of exclusion in which there are a series of disorders needing to be precluded, including atherosclerosis, autoimmune disease, Down’s syndrome, cerebrovascular lesions after head irradiation, and so on, and it relies on the neuroimaging findings such as cerebral angiography [1,2]. As for treatment, the main focus at present is to improve an MMD patient’s hemodynamic status to reduce the risk of stroke through revascularization surgery [1,2]. In order to optimize diagnostic and therapeutic strategies and to improve the current clinical situation, studies on the etiology of MMD are urgent. It has been reported that angiogenesis was inhibited in MMD *RNF213* R4810K carriers under hypoxic conditions [9]. However, there is a lack of research on the pathogenesis underlying the involvement of hypoxia in MMD. Our study found that four hypoxia-immune genes, namely, *AKT1*, *CLDN3*, *ISG20*, and *TGFB2*, were promising candidates for diagnostic and therapeutic targets in MMD by combining bioinformatics methods including immune infiltration, high-throughput sequencing, and machine learning. We further enhanced the reliability of these results with the validation of external data.

*ISG20* encodes the interferon-stimulated gene 20 kDa protein (ISG20) which is a member of the DEDD exonucleases domain superfamily with strong RNase properties, and plays a crucial role in the pathophysiological processes of inhibiting a broad spectrum of viruses, mainly via directly degrading viral RNAs [28]. Additionally, it was also reported that both in vivo and in vitro, the up-regulation of ISG20 significantly promoted angiogenesis in liver cancer cells [29]. In another in vitro angiogenic assay, ISG20 ExoII, a dominant negative mutant of ISG20, inhibited angiogenesis in human umbilical vein endothelial cells [30]. These results provide indirect support for our findings that *ISG20* up-regulation found in both the training and validation sets might be involved in the pathogenesis of MMD via affecting angiogenesis. In addition, the KEGG signaling pathways in which *ISG20* is mainly involved, including leishmania_infection, graft-versus-host_disease, and antigen_processing_and_presentation, are closely related to immune mechanisms [31,32,33]. These results also implied that *ISG20* might also contribute to MMD via immune-related mechanisms.

Claudin 3 (CLDN3) encoded by *CLDN3* is a member of the claudin transmembrane protein family, which polymerizes to form the backbone of tight junction strands [34]. Based on the training and validation set expression level assessments, we found that CLDN3 was up-regulated in MMD. The overexpression of CLDN3 can lead to a decrease in or even loss of cell–cell adhesion, resulting in the promotion of epithelial–mesenchymal transition (EMT), which is implicated in the pathophysiology of numerous types of cancers [35,36]. Pathologic studies of MMD have revealed a thickening of the intima and vascular smooth muscle cell migration in the terminal internal carotid artery [37,38]. Altered CLDN3 expression levels may be associated with the vascular changes in MMD. In addition, CLDN3 is also involved in the composition of the blood–brain barrier [39]. Drugs targeting it may be more effective in central nervous system diseases like MMD. Interestingly, the toll-like_receptor signaling pathway involved in *CLDN3* has an essential position in the innate immune system [40]. This finding suggests that innate immunity might be associated with MMD.

Transforming growth factor beta 2 (TGFB2), also known for its involvement in the regulation of endothelial cell homeostasis, carries significant weight in EMT [41]. The disorders associated with TGFB2 signaling can lead to vascular pathology, resulting in cardiovascular and fibrotic diseases [41,42]. Previous studies have shown that the overexpression of TGFB2 promotes the process of EMT and is involved in angiogenesis [41,43]. Therefore, disturbances in TGFB2 expression levels may be associated with pathologic vascular alterations and angiogenesis in MMD. *TGFB2* was also enriched into many immune-related signaling pathways, including leishmania_infection, graft-versus-host_disease, and allograft_rejection signaling pathways [31,32,44]. Further studies of related signaling pathways in MMD might be helpful in understanding the underlying pathogenesis.

*AKT1* encodes ATK1 which is one of the three members of human AKT serine–threonine protein kinase family, known as protein kinase B family [45]. The activation of AKT1 can inhibit apoptosis, positively regulate cell proliferation, as well as promote cell migration, and it can exert a regulatory influence in a range of cancers [45,46]. However, it was also reported that the down-regulation of AKT1 was involved in squamous cell carcinomas (SCC) and human papillomavirus (HPV) infection [47]. O’Shaughnessy et al. found that before papilloma formation or tumorigenesis, cutaneous HPV8 down-regulated epidermal AKT activity by down-regulating AKT1 isoenzyme levels, leading to changes in cutaneous differentiation that might weaken the epidermis and thus increase the capacity for viral release [47]. In our study, AKT1 expression was significantly down-regulated in the MMD group compared to the control group. However, the relation between AKT1 and MMD is still largely blank. Given the disruption of the epidermal barrier by AKT1 inhibition, we inferred that in MMD, AKT1 down-regulation might be involved in the pathogenesis by affecting the integrity of the vascular wall. Additionally, the results of GSEA showed that *AKT1* was also extensively involved in the leishmania_infection, graft-versus-host_disease, allograft_rejection, and antigen_processing_and_presentation signaling pathways, which further suggests that these pathways might be closely related to the etiopathogenesis of MMD.

Previous studies have found that the infiltrating levels of immune cells might be higher in MMD [25,48,49]. We also found that the level of immune cell infiltration was usually higher in the MMD group, and in particular, there was a significant difference in the level of expression of macrophages, immature B cells, monocytes, and helper T cells type 17 between the MMD group and the control group by analyzing data from public databases. Building on this, we performed histological analysis on the STA tissues of both the MMD group and the control group to validate the presence of vascular immune cell infiltration in Moyamoya disease. The results showed significant CD56 staining in the intima and media layers of the STA tissues from patients with MMD. These findings suggest a considerable increase in immune cell infiltration within the STAs of MMD patients.

In this study, we identified ritodrine, (+)-isoprenaline, terbutaline, Colforsin, Isoxsuprine, dobutamine, metanephrine, ethaverine, 16,16-dimethylprostaglandin E2, and etilefrine—10 drugs that may potentially play a role in the treatment of MMD through the four key hypoxia-immunity-related gene pathways identified above. However, our findings are the result of standard data analysis, where the primary goal is to achieve biological statistical significance. Additionally, the clinical translation of these drugs is challenging, and there is still no in vitro–in vivo validation of their role in the treatment of MMD. We hope these findings will inspire pharmaceutical practitioners in their quest to develop drugs for the treatment of MMD.

In addition, the size of the datasets is limited this study. And although samples of controls were obtained from patients with aneurysms or epilepsy, which were considered acceptable in studying MMD, the results were still partially affected. This study focused solely on STA samples, and future work could benefit from including peripheral WBC samples to further validate the findings. In vivo and in vitro experiments in a large number of MMD patients of different ethnicities will be needed to identify its etiology.

## 5. Conclusions

In this study, we found that *AKT1*, *CLDN3*, *ISG20*, and *TGFB2* were identified as the feature hypoxia-immune genes relevant to MMD through a bioinformatics approach, which combined high-throughput sequencing, immune infiltration, machine learning, and external validation, and that these genes could provide new perspectives for the diagnosis of MMD.

## Data Availability

The original contributions presented in the study are included in the article; further inquiries can be directed to the corresponding author.
